# P34 Gentamicin labels: improving the timing of gentamicin levels and reducing delay in dose administrations

**DOI:** 10.1093/jacamr/dlad066.038

**Published:** 2023-06-14

**Authors:** Muhammad Zakwan Zakariya, Esperanza Palenzuela, Rebekah Sutherland, Khalida Ann Lockman

**Affiliations:** NHS Lothian, Scotland; NHS Lothian, Scotland; NHS Lothian, Scotland; NHS Lothian, Scotland

## Abstract

**Background:**

Due to its narrow therapeutic index and significant risks of ototoxicity and nephrotoxicity, gentamicin requires meticulous prescribing, administration and monitoring. Despite readily available guidelines, gentamicin use continues to be associated with errors, with recent Datix reports within NHS Lothian recording incidents such as incorrectly prescribed doses or frequencies, missed levels, and delayed or omitted doses. In response to this, an audit was undertaken to investigate errors in gentamicin use within the Acute Medical Unit (AMU) at the Royal Infirmary of Edinburgh and evaluate the barriers to adherence to guidelines to unearth possible areas for improvement.

**Methods:**

The initial audit studied patients prescribed gentamicin in AMU within a 3-week period, excluding those who received prophylactic or single one-off doses of gentamicin. The following parameters were examined both on their paper and electronic prescribing charts: (i) dose, (ii) dosing interval/frequency, (iii) time of level sampling, and (iv) administration times of the first and second gentamicin doses. After identifying issues with delayed gentamicin levels and subsequent dose administration times, we introduced the use of gentamicin labels in each AMU bay, in hopes of increasing staff awareness towards patients on gentamicin, thereby promoting timely level sampling and dose administration. The aforementioned parameters were re-audited for a further 3 weeks following this intervention.

**Results:**

The baseline audit highlighted that only 23/31 (74%) patients had levels taken within the recommended window, and 23/31 (74%) received their second gentamicin doses at the right time. Following the introduction of the gentamicin labels, the second audit observed a slight improvement, with 31/40 (78%) patients having their levels taken within the recommended window, and 30/40 (75%) patients receiving their subsequent doses at the right interval. Noteworthily, in both the pre- and post-intervention groups, the majority of patients who had their second doses delayed received their first doses in the emergency department (ED) prior to admission to AMU.

**Conclusions:**

In addition to improving staff awareness towards patients on gentamicin, a further overarching issue needs to be addressed: the delays in gentamicin administration which more commonly occurs for patients who transition from ED into AMU. Further interventions should therefore target improving the handover of time-critical medicines between ED and AMU.

